# Glacier retreat alters downstream fjord ecosystem structure and function in Greenland

**DOI:** 10.1038/s41561-023-01218-y

**Published:** 2023-06-29

**Authors:** Lorenz Meire, Maria Lund Paulsen, Patrick Meire, Søren Rysgaard, Mark James Hopwood, Mikael Kristian Sejr, Alice Stuart-Lee, Koen Sabbe, Willem Stock, John Mortensen

**Affiliations:** 1 Royal Netherlands Institute for Sea Research, Department of Estuarine and Delta Systems, Yerseke, the Netherlands; 2 Greenland Climate Research Centre, Greenland Institute of Natural Resources, Nuuk, Greenland; 3 Arctic Research Centre, Aarhus University, Aarhus, Denmark; 4 Marine Microbiology, Bergen University, Bergen, Norway; 5 Ecosystem Management Research Group, Department of Biology, University of Antwerp, Antwerpen, Belgium; 6 Centre for Earth Observation Science, CHR Faculty of Environment, Earth, and Resources, University of Manitoba, Winnipeg, Manitoba Canada; 7 School of Ocean Science and Engineering, Southern University of Science and Technology, Shenzhen, China; 8 Laboratory of Protistology & Aquatic Ecology, Ghent University, Ghent, Belgium

**Keywords:** Marine biology, Carbon cycle, Physical oceanography

## Abstract

The melting of the Greenland Ice Sheet is accelerating, with glaciers shifting from marine to land termination and potential consequences for fjord ecosystems downstream. Monthly samples in 2016 in two fjords in southwest Greenland show that subglacial discharge from marine-terminating glaciers sustains high phytoplankton productivity that is dominated by diatoms and grazed by larger mesozooplankton throughout summer. In contrast, melting of land-terminating glaciers results in a fjord ecosystem dominated by bacteria, picophytoplankton and smaller zooplankton, which has only one-third of the annual productivity and half the CO_2_ uptake compared to the fjord downstream from marine-terminating glaciers.

## Abstract (web summary)

Glacier retreat in Greenland not only changes the primary productivity of downstream fjord ecosystems but also the ecosystem structure and functioning, according to seasonal sampling of two downstream fjords.

## Main

Greenland’s fjords play an essential role as pathways connecting the Greenland Ice Sheet to the surrounding ocean harbouring productive ecosystems acting as carbon sinks^1^ with socio-economic important fisheries^2^.

The upwelling of subglacial meltwater supplies nutrients to the surface layer leading to higher production in fjords impacted by marine-terminating glaciers compared to fjords solely impacted by land-terminating glacier runoff^2,3^. The role of glaciers in downstream marine food web dynamics, however, remains poorly understood. As most marine-terminating glaciers show evidence of accelerated retreat and becoming land-terminating, an understanding of the role of different glacier types on downstream fjord ecosystem dynamics is needed to apprehend the impacts of climate change on the food web and associated carbon sinks. Here, we present data collected in two fjords adjacent to the Greenland Ice Sheet which show that glacier retreat will not only impact primary productivity but also the ecosystem structure and function.

**Fig. 1:**
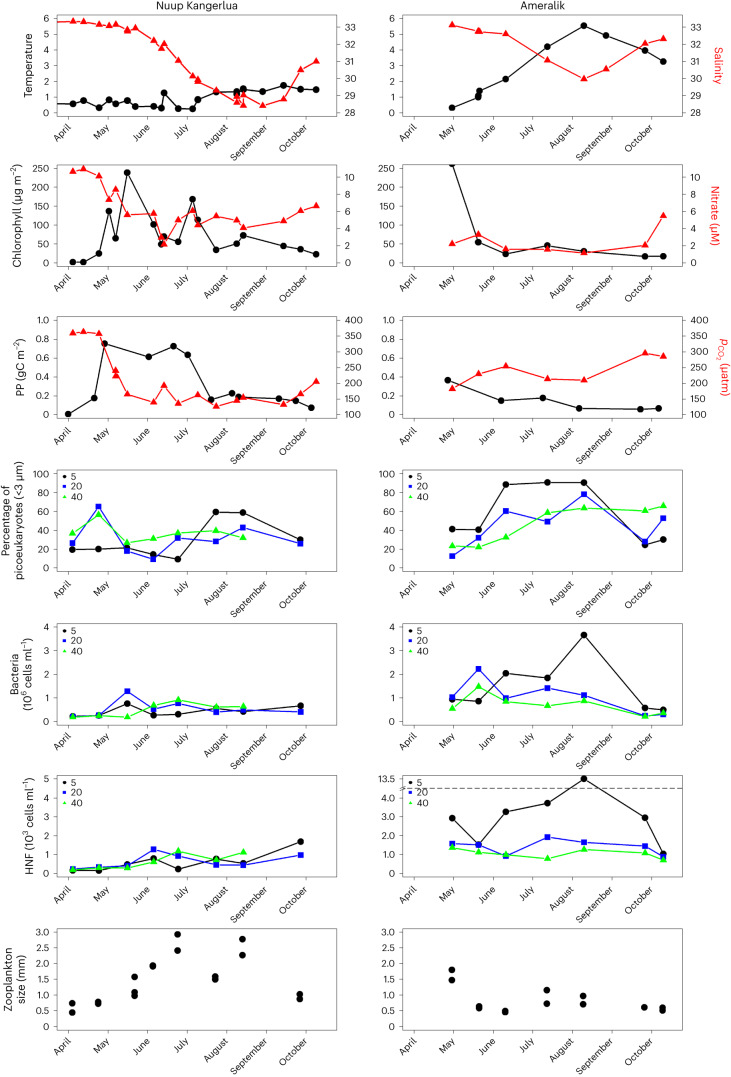
Seasonal dataset from a station in fjord impacted by land-terminating (Ameralik) and marine-terminating (Nuup Kangerlua) glaciers. Seasonal data from 2016 at station in Nuup Kangerlua and Ameralik: average temperature and salinity, integrated chlorophyll biomass (mg cholorophyll *a* m^−2^) and average nitrate (µM), integrated primary production (PP; all upper 40 m) and $p_{{\mathrm{CO}}_2}$ (µatm) at 1 m; % picoeukaryotes <3 µm in total phytoplankton biomass, bacterial abundance (10^6^ cells ml^−1^), abundance of HNF (10^3^ cells ml^−1^) at 5 m (black), 20 m (blue) and 40 m (green); and average mesozooplankton size (mm) in the upper 100 m.

**Extended Data Fig. 1:**
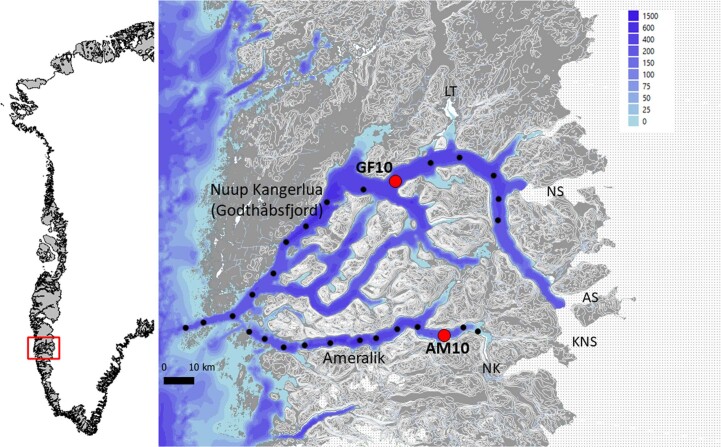
Map of the study area. Overview map of Greenland with indication of the study site, Ameralik and Nuup Kangerlua (Godthåbsfjord). The two focus stations (GF10 and AM10) are indicated on the map together with glaciers (NS: Narsap Sermia, Kangiata Nunaata Sermia (KNS), Akullersuup Sermia (AS)), incoming rivers (Lake Tasersuaq (LT), Naujat Kuat River (NK)).

**Extended Data Fig. 2:**
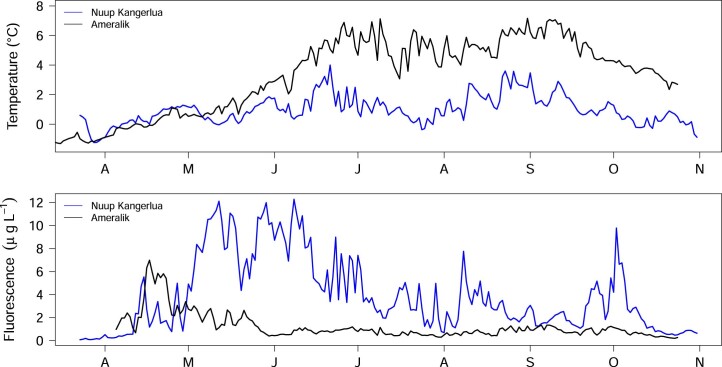
Temperature and fluorescence mooring data from the study sites. Seasonal evolution during 2018 of temperature (°C) and fluorescence (calibrated to chlorophyll-a (µg L^−1^)) in Nuup Kangerlua (blue) and Ameralik (black) based on a mooring at 5 m depth.

**Extended Data Fig. 3:**
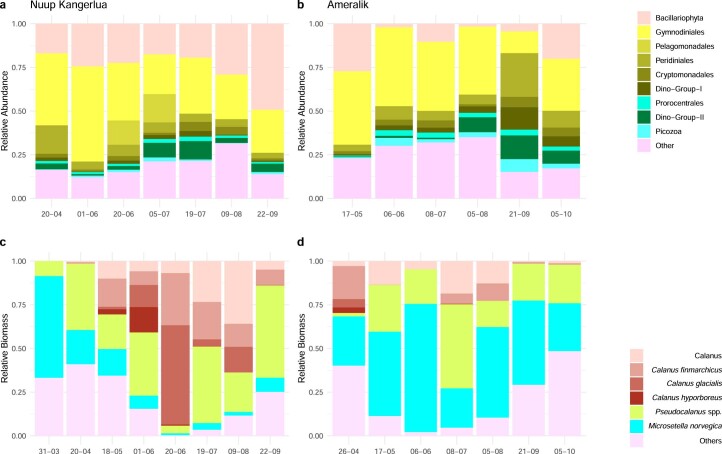
Protist and mesozooplankton species composition during 2016. Seasonal evolution during 2016 of protist community composition based on amplicon sequencing (**a, b**) and mesozooplankton species composition (**c, d**) in vertical net tows in Nuup Kangerlua (GF10) and Ameralik (AM10).

Seasonal samples were collected at two stations in the neighbouring fjords: Nuup Kangerlua and Ameralik, impacted predominantly by marine- and land-terminating glaciers, respectively (Fig. 1 and Extended Data Fig. 1). Both fjords show pronounced freshening in the surface layer from June to September (Fig. 1) due to the large input of meltwater. A large difference in surface temperature persists from spring to autumn with temperatures in Nuup Kangerlua being 3–4 °C lower than in Ameralik from July to October. In spring, both fjords display a pronounced spring bloom with a high phytoplankton biomass (200–250 mg chlorophyll *a* m^−2^) and distinct drawdown of nitrate in the upper 40 m (Fig. 1). After spring, a divergent pattern in phytoplankton abundance and community composition is observed (Fig. 1). In Nuup Kangerlua with marine-terminating glaciers, larger phytoplankton (>5 µm) remain abundant and metabarcoding data reveal the continuous presence of *Bacillariophyta* (diatoms) as observed in a North Greenland fjord^4^. Chlorophyll *a* in the photic zone is higher and primary production measurements show rates of ~200 to ~800 mgC m^−3^ d^−1^ from June to August (Fig. 1 and Extended Data Fig. 2). The renewed supply of nitrate due to subglacial discharge and excess silicate in the surface layers are probably the main drivers for diatom persistence in Nuup Kangerlua^5^. In Ameralik, impacted only by a land-terminating glacier, phytoplankton biomass is lower during summer and dominated by picophytoplankton in the warmer surface layers (with abundances >30,000 cell ml^−1^) (Fig. 1 and Extended Data Fig. 3). The substantially lower supply of nitrate to the surface waters results in a lower nitrate concentration in the upper 40 m and consequently lower primary productivity with rates <200 mgC m^−3^ d^−1^. The annual pelagic primary production in 2016 was estimated as to be three times higher in Nuup Kangerlua (~90 gC m^−2^ yr^−1^) compared to Ameralik (~30 gC m^−2^ yr^−1^). The large difference in productivity between the two regions is also reflected in the partial pressure of CO_2_ ($p_{{\mathrm{CO}}_2}$). After the spring boom, an undersaturation in $p_{{\mathrm{CO}}_2}$ is observed in both fjord systems but while in Nuup Kangerlua the $p_{{\mathrm{CO}}_2}$ concentration drops to 150 ppm in the upper meter, in Ameralik concentrations remain ~250 ppm. The difference results in a CO_2_ uptake of ~42 gC m^−2^ in Nuup Kangerlua (GF10) compared to ~24 gC m^−2^ in Ameralik (AM10) from April to September.

Differences in the functioning of Greenland’s fjords not only impact primary producers but also strongly influence the abundance and activity of heterotrophic microorganisms. Following the spring bloom, an increase in bacterial abundance was observed in both fjords (Fig. 1). From June to September, the bacterial community developed very differently in the two fjords, with an up to ten times higher bacterial abundance in the surface waters of Ameralik than in Nuup Kangerlua. A surface measurement from Ameralik in August showed a bacterial abundance of 3.7 × 10^6^ cells ml^−1^, which is high compared to earlier observations in the Arctic^6^. Bacterial production was previously found to be elevated in turbid meltwater plumes in Young Sound, northeastern Greenland^7,8^ and Hornsund, Svalbard^9^, indicating that heterotrophic processes are more important in glacial river-influenced areas. Whilst meltwater could provide a source of labile carbon to bacterial communities in glacier fjords^10^, concentrations are lower compared to coastal waters and bacterial communities probably rely primarily on autochthonous carbon sources^11,12^. Light limitation of photosynthesis due to high turbidity in the inner part of Ameralik gives bacteria in surface waters a further competitive edge for nutrients over phytoplankton^13^. Heterotrophic nanoflagellates (HNF) are protozooplankton grazing on bacteria and picophytoplankton^14^ and therefore key organisms in transferring production to higher trophic levels. Throughout the season, HNF are more abundant in Ameralik, suggesting a tight grazing control on both picophytoplankton and bacteria (Fig. 1).

**Extended Data Fig. 4:**
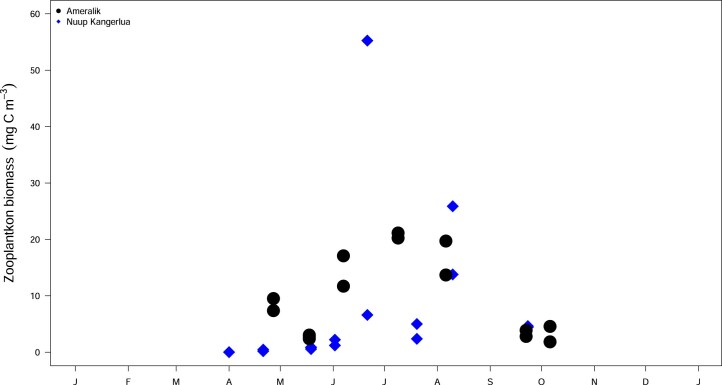
Change in zooplankton biomass in two study sites throughout the season. Evolution of zooplankton biomass (mgC m^−2^) in station in Nuup Kangerlua (blue) and Ameralik (black) during 2016.

Mesozooplankton in the upper 100 m differ in size spectrum and species composition between the two fjords (Fig. 1), although there were no consistent differences in total biomass between the stations (Extended Data Fig. 4). During summer, larger *Calanus* species dominate the zooplankton community in Nuup Kangerlua while smaller species like *Microsetella norvegica* are more dominant in Ameralik (Extended Data Fig. 3). *M. norvegica* is a small copepod frequently associated with aggregates with a temperature optimum between 6 and 8 °C (ref. ^15^) and has previously been observed to be dominant at the mouth of both fjords^15^. The higher abundance of smaller phytoplankton, combined with higher surface temperature in Ameralik, probably benefits this small copepod. The presence of larger boreo-arctic species observed in Nuup Kangerlua, which probably feed on large chains of diatoms, suggests different nutritional values which could play a major role in transfer to higher trophic levels, especially as they are the preferred food type for capelin (*Mallotus villosus*) in the fjord^16^.

**Fig. 2:**
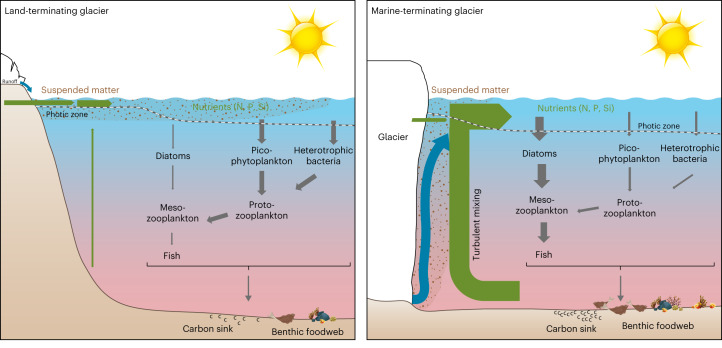
Conceptual figure of the impact of different glacier types (land- versus marine-terminating) on oceanography and ecosystem functioning. Illustration of how different glacier types (land- versus marine-terminating) impact oceanography and ecosystem functioning. Meltwater runoff of land-terminating glaciers results in stratified, turbid inner-fjord surface layer characterized by a low productivity dominated by picophytoplankton and bacteria. These are primarily grazed by smaller protozooplankton. Fjords with marine-terminating glaciers are characterized by upwelling of nutrient-rich deep water by subglacial discharge plumes stimulating higher phytoplankton productivity and diatoms which are grazed by larger mesozooplankton, potentially resulting in more efficient transfer to higher trophic levels.

Melting of the ice sheet will have major ramifications for Greenland’s fjords (Fig. 2). At marine-terminating glaciers, subsurface release of meltwater stimulates diatom blooms, which in turn form a food source for many of the larger zooplankton species. Conversely, large inputs of turbid meltwater from land-terminating glaciers result in a strongly stratified, low-light environment characterized by higher temperatures, lower phytoplankton productivity and increased abundances of bacteria, picophytoplankton and HNF. For marine-terminating glaciers that continue retreating^17^, this will result in changes in the hydrography, carbon cycling and ecological functioning of the respective fjords. Upon the transition of marine-terminating glaciers into land-terminating systems, a lack of direct glacier ice discharge into the fjord will cause further warming of the surface layer and, when subglacial discharge ceases, reduced circulation in the fjord will result in a lower nutrient supply. A substantial fraction of Greenland’s fjords therefore face the prospects of a long-term succession in ecosystem structure from diatom-dominated to more pico-sized-phytoplankton- and bacteria-dominated ecosystems. These transitions will lead to changes in both the CO_2_ uptake potential and quantity and quality of fjord productivity with cascading to higher trophic levels.

## Methods

Seasonal samples with monthly resolution were collected in Nuup Kangerlua and Ameralik in 2016 at a station in each fjord (GF10 and AM10). Nuup Kangerlua is a large fjord system located in West Greenland with a length of ~190 km covering an area of 2,013 km^2^ (Extended Data Fig. 1). Three marine-terminating glaciers are located in the catchment: Kangiata Nunaata Sermia (KNS), Akullersuup Sermia (AS) and Narsap Sermia (NS), all delivering glacial ice and meltwater to the fjord^18^. Hydrological simulations for the period 1991–2012 estimate an annual meltwater input of 20 km^3^ yr^−1^ and a solid ice discharge of ~8 km^3^ yr^−1^ (refs. ^19,20^). Ameralik is a fjord just south of Nuup Kangerlua with a total area of 350 km^2^ and length of ~70 km. The fjord is fed by a land-terminating glacier, the Naujat Kuat River (64° 12′ 37.5′′ N, 50° 12′ 31. 0′′ W). In 2012, discharge from the glacial river was estimated as ~0.8 km^3^ yr^−1^ (ref. ^21^). Both fjords have a comparable climate and share the same oceanographic boundary conditions allowing the impacts of different glacier types to be investigated.

Salinity and temperature depth profiles were recorded using a CTD instrument (Seabird SBE19plus) equipped with additional sensors for fluorescence (Seapoint Chlorophyll Fluorometer), turbidity (Seapoint) and Photosynthetic Active Radiation (Biospherical QSP-2350L Scalar sensor). Partial pressure of carbon dioxide (*p*_CO2_) was measured in situ using the HydroC Carbon Dioxide Sensor (Contros) below the surface (1 m). The HydroC sensor was equilibrated for 2–5 min until a stable reading was obtained. The relative standard deviation of the *p*_CO2_ measurement has been estimated to be 1% (ref. ^22^). Mesozooplankton were collected in the upper 100 m with a 50 μm mesh WP2 net using duplicate vertical hauls and counted down to genus level and developmental stage. Water samples from discrete depths (1, 5, 10, 20, 30, 40 and 50 m) were collected using a 5 l Niskin. To calibrate the fluorescence sensor, water samples (0.5 l) were filtered through 25 mm GF/F filters (Whatman, nominal pore size 0.7 µm) for chlorophyll *a* analysis. Filters were placed in 10 ml of 96% ethanol for 18 to 24 h and chlorophyll fluorescence in the filtrate was analysed using a fluorometer (Trilogy, Turner Designs) before and after addition of 200 μl of 1 M HCl solution to correct for the presence of phaeopigments. Subsamples (10 ml) for nutrients were filtered through 0.45 µm filters (Q-Max GPF syringe filters) and directly frozen at −20 °C until analysis. Nutrients were measured using standard colorimetric methods on a Seal QuAAtro autoanalyser. Additionally, nitrate profiles were collected using a SUNA V2 (SAtlantic, Seabird). Nitrate measurements were corrected for salinity and temperature using the equations in ref. ^23^. These continuous measurements were validated with discrete samples at different depths. Samples for the abundances of bacteria, HNF and phytoplankton were collected directly from the Niskin and fixed with glutaraldehyde (0.5% final concentration) and kept frozen at −80 °C until analysis. Abundances were determined on an Attune flow cytometer (Applied Biosystems by Life Technologies) with a syringe-based fluidic system and a 20 mW 488 nm (blue) laser. Heterotrophic cells were stained with SYBR Green I DNA stain and identified on the basis of their red and green fluorescence and sidescatter. Increase in the ratio between high nucleic acid (HNA) bacteria and low nucleic acid (LNA) bacteria, is used as an indicator for bacterial activity. Phytoplankton populations were discriminated on the basis of their pigments and the biomass of three size groups was calculated using a carbon conversion factor of 2.59 pgC cell^−1^ for picophytoplankton^23,24^, 7.37 pgC cell^−1^ for small nanophytoplankton (mean diameter 4 ± 0.5 µm) and 58.98 pgC cell^−1^ for large nanophytoplankton (mean diameter 8 ± 0.5 µm) (ref. ^25^).

For DNA analysis, filter samples were taken through 0.45 μm nitrocellulose filters (Millipore, volume of 500–1,000 ml). The V4 region of the 18S ribosomal RNA gene was amplified and sequenced as in ref. ^26^, using the primer set TAReuk454FWD1 (5′-CCAGCASCYGCGGTAATTCC-3′) and TAReukREV3 (5′-ACTTTCGTTCTTGATYRA-3′). The PCR mixture which had a final volume of 25 µl, contained 1 µl of template DNA, 200 µM of each deoxyribonucleotide triphosphate, 0.4 µM of each primer, 0.25 U of Fast Start High Fidelity Taq polymerase (Roche). PCRs (35–40 touch-down cycles of 1 min at 94 °C, 1 min at 57–52 °C and 3 min at 72 °C, with an initial denaturing step of 5 min at 94 °C and a final step of 20 min at 72 °C) were run in duplicate to reduce stochasticity. The PCR products were purified with Agencourt AMPure XP beads. The amplicon libraries were barcoded using the NEXTERA xt DNA kit (Illumina) following manufacturer’s instructions and purified using Agencourt AMPure XP beads. Libraries were sequenced on a 300 base pair paired-end Illumina MiSeq platform. The high throughput sequence data are available in the NCBI SRA BioProject database under accession PRJNA894377. The 18S Illumina MiSeq data were processed using the default dada2 pipeline^27^ (v.1.14.1) in R. Primers were removed after which the reads were filtered, discarding any reads with more than two expected errors. Amplicon Sequence Variants (ASV) were estimated using the Illumina-specific error model. Bimeric sequences were removed using the consensus method within the dada2 package. The PR2 database^28^ (v.4.14.0) was used for taxonomic assignment of the ASV. Phyloseq^29^ (v.1.30.0) was used to further process the ASVs afterwards. The full dataset is available as Supplementary Data.

Primary production rates were calculated according to methodology in ref. ^30^. Solar irradiance was obtained from the meteorological survey in Nuuk (Meteorological station 522, Asiaq Greenland Survey) for the 14-day period. Annual production was estimated by calculating daily productivity over the entire year assuming that light extinction and PI curves remain the same in the 2 week period before and after the sampling dates. Air–sea CO_2_ exchange was calculated according to methodology in ref. ^30^. Wind data were obtained from the meteorological station in Nuuk.

Processing of data was done in the open-source programming language R. Interpolation of the data and contour plots were produced using the OceanView package^31^.

## Online content

Any methods, additional references, Nature Portfolio reporting summaries, source data, extended data, supplementary information, acknowledgements, peer review information; details of author contributions and competing interests; and statements of data and code availability are available at 10.1038/s41561-023-01218-y.

## Supplementary information

Supplementary Data Seasonal evolution of protist community composition (in percentage) based on amplicon sequencing.

## Data Availability

The raw data used in this study can be found in the Figshare data repository (10.6084/m9.figshare.23153741).
